# A review of technological innovations leading to modern endovascular brain aneurysm treatment

**DOI:** 10.3389/fneur.2023.1156887

**Published:** 2023-04-11

**Authors:** David C. Lauzier, Anna L. Huguenard, Anja I. Srienc, Samuel J. Cler, Joshua W. Osbun, Arindam R. Chatterjee, Ananth K. Vellimana, Akash P. Kansagra, Colin P. Derdeyn, Dewitte T. Cross, Christopher J. Moran

**Affiliations:** ^1^Mallinckrodt Institute of Radiology, Washington University in St. Louis School of Medicine, St. Louis, MO, United States; ^2^Department of Neurological Surgery, Washington University in St. Louis School of Medicine, St. Louis, MO, United States; ^3^Department of Neurology, Washington University in St. Louis School of Medicine, St. Louis, MO, United States; ^4^Department of Neurointerventional Surgery, California Center of Neurointerventional Surgery, San Diego, CA, United States; ^5^Department of Radiology, University of Iowa School of Medicine, Iowa City, IA, United States

**Keywords:** aneurysm, flow diversion, coil embolization, endovascular, stent

## Abstract

Tools and techniques utilized in endovascular brain aneurysm treatment have undergone rapid evolution in recent decades. These technique and device-level innovations have allowed for treatment of highly complex intracranial aneurysms and improved patient outcomes. We review the major innovations within neurointervention that have led to the current state of brain aneurysm treatment.

## Introduction

**Figure 1:**
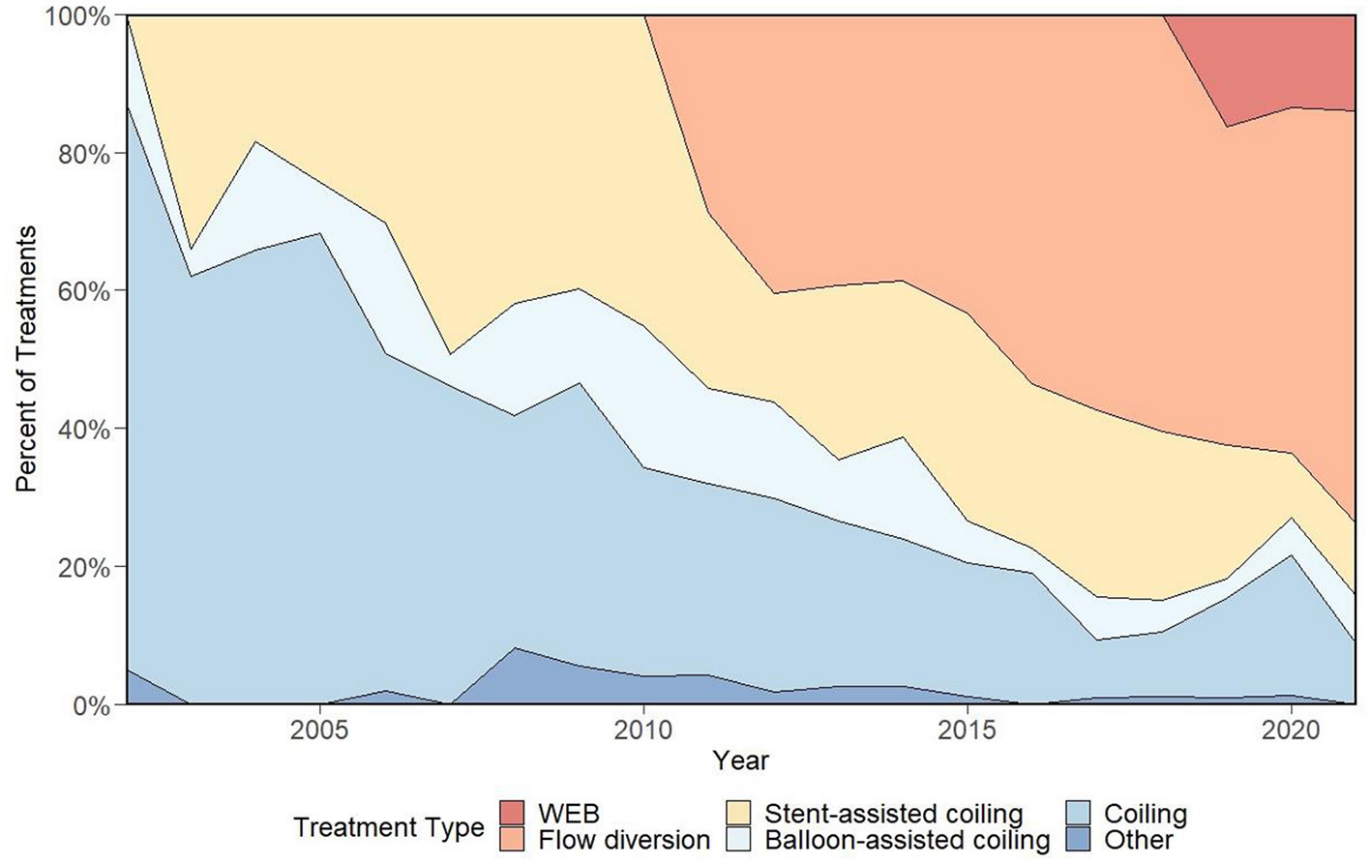
Representation of device selection for elective aneurysm treatments at our center over a 20-year period, which demonstrates a rapid adoption of flow diversion following its arrival and early signs of a similar trend with the WEB device. Figure adapted from prior work (6).

The main goals of intracranial aneurysm treatment are to prevent rupture, thrombosis, or symptoms of mass effect (1, 2). Endovascular treatments have provided a viable alternative to open surgical strategies for aneurysms in several cerebrovascular territories (3–5). In the present work, we discuss the evolution of neurointerventional aneurysm treatment from its origins to the modern era, review state-of-the art strategies for the treatment of intracranial aneurysms, and describe technical factors leading to the adoption of certain strategies and the phasing out of others. Additionally, we provide details regarding the case volume for various treatment approaches at our center to display the integration of novel treatment strategies into an academic neurointerventional practice and showcase individual practice variation over time (Figure 1). Treatments discussed focus on devices approved by the United States Food and Drug Administration (FDA), however other important devices are also included in our discussion (Table 1).

**Table 1:** Major representative endovascular devices used for brain aneurysm treatment.

**Device**	**Manufacturer and year of FDA approval**	**Material**	**Detachment**
**Coils**			
Target (Guglielmi) detachable coil	Target, 1995	Bare platinum	Electrolytic
Micrus microcoil	Micrus, 2001	Bare platinum	Mechanical (using resistive heating)
HydroCoil embolic system	Microvention, 2002	Hydrogel-coated bare platinum coils	V-grip detachment controller with self-contained integral power supply
Orbit detachable coil	Codman, 2003	Bare Platinum	Mechanical/Hydraulic
Matrix bioactive coil	Boston Scientific/Target, 2003	Polyglycolic acid-coated bare platinum coils	Electrolytic
Cerecyte coil	Micrus, 2004	Platinum coils with polyglycolic acid running through lumen of primary platinum wind	Mechanical (using resistive heating)
Orbit Galaxy coil	Cerenovus, 2010	Bare platinum	Mechanical (using resistive heating)
PC400 coil	Penumbra, 2012	Bare platinum	Mechanical
Codman Trufill coil	Johnson and Johnson, 2013	Bare platinum	Mechanical (hydraulic)
Axium	Medtronic, 2014	Bare platinum	Mechanical
Barricade coil	Balt, 2015	Bare platinum	Electrolytic
Axium prime	Medtronic, 2017	Bare platinum	Mechanical
Optima coil	Balt, 2020	Bare platinum	Thermal
**Stents**			
Device	Manufacturer and Year of FDA Approval	Material	Delivery System (size)
Neuroform stent	Boston Scientific/Target, 2002	Nitinol, open-cell design	0.027-inch microcatheter
Enterprise stent	Cordis Neurovascular, 2007	Nitinol, closed-cell design coated with insulating polymer (Parylene C)	0.021-inch microcatheter
LVIS, LVIS Jr	Microvention, 2018	Nitinol, closed-cell braided design	0.021-inch, 0.0165-inch microcatheter, respectively
LVIS EVO	Microvention	Nitinol, closed-cell braided design	0.0165-inch microcatheter
Neuroform Atlas	Stryker Neurovascular, 2019	Nitinol, laser-cut open-cell design	0.0165-inch microcatheter
**Flow diverters**			
Device	Manufacturer and year of FDA approval	Material	Delivery catheter size
Pipeline Embolization Device	Medtronic Neurovascular, 2011	36 cobalt-chromium strands, 12 platinum-tungsten strands woven in 3:1 ratio	0.027-inch microcatheter
Pipeline Flex Embolization Device	Medtronic Neurovascular, 2018	36 cobalt-chromium strands, 12 platinum-tungsten strands woven in 3:1 ratio	0.027-inch microcatheter
Pipeline Flex with Shield	Medtronic Neurovascular, 2021	36 cobalt-chromium strands, 12 platinum-tungsten strands woven in 3:1 ratio, phosphorylcholine polymer surface coating	0.027-inch microcatheter
Pipeline Vantage	Medtronic Neurovascular	36 or 48 cobalt-chromium strands, 12 or 16 platinum-tungsten strands woven in 3:1 ratio, phosphorylcholine polymer surface coating	0.021-inch or 0.027-inch microcatheter
FRED	Microvention, 2019	Self-expanding braided 66 nitinol wires	0.027-inch microcatheter
FRED Jr	Microvention, 2019	Self-expanding braided 54 nitinol wires	0.021-inch microcatheter
FRED X	Microvention	Self-expanding braided 54 or 66 nitinol wires, protective nano-polymer hydration layer	0.021-inch or 0.027-inch microcatheter
Surpass Streamline	Stryker Neurovascular, 2018	72 or 96 cobalt-chromium wires	0.053-inch intermediate catheter, passed over 0.014-inch microwire
Surpass Evolve	Stryker Neurovascular, 2020	64 cobalt-chromium wires	0.027-inch microcatheter
Silk	Balt Extrusion	48 braided nitinol wires	0.021-inch or 0.025-inch microcatheter
Silk Vista	Balt Extrusion	48 braided nitinol wires	0.021-inch or 0.025-inch microcatheter
Silk Vista Baby	Balt Extrusion	48 braided nitinol wires	0.017-inch microcatheter
**Other**			
Device	Manufacturer and year of FDA approval	Material	Delivery catheter size
Onyx HD-500	ev3, 2007	20% Ethylene vinyl alcohol copolymer dissolved in DMSO with suspended micronized tantalum powder	0.014-inch microcatheter
PulseRider Device	Cerenovus, 2018	Nitinol, Y or T-shape	0.021-inch microcatheter
pCONUS	Phenox	Nitinol, radially-flaring distal petals	0.021-inch microcatheter
pCONUS 2	Phenox	Nitinol, radially-flaring distal petals	0.021-inch microcatheter
pCONUS 2-HPC	Phenox	Nitinol, radially-flaring distal petals with hydrophilic coating	0.021-inch microcatheter
WEB	Microvention, 2018	Nitinol wires with platinum coil, now single layer of mesh	0.017, 0.021, 0.027, or 0.033-inch microcatheter
Contour	Cerus	Dual-layered structure of 2 × 72 nitinol wires	0.021-inch or 0.027-inch microcatheter
**Balloons**			
Device	Manufacturer and Year of FDA Approval	Lumen type	DMSO compatible?
Commodore	Cordis Neurovascular, 1999	Single lumen	No
Equinox	Micro Therapeutics, 2000	Single lumen	No
Ascent	Micrus, 2008	Single lumen	No
HyperGlide	Medtronic Neurovascular, 2010	Single lumen	Yes
HyperForm	Medtronic Neurovascular, 2010	Single lumen	Yes
Scepter	Microvention, 2012	Dual lumen	Yes
TransForm	Stryker Neurovascular, 2013	Single lumen	No
Eclipse	Balt, 2019	Dual lumen	Yes

Year of FDA approval provided for devices available in the United States.

Similar to device evolution, there have been dramatic changes in device manufacturers with many mergers and buyouts. For example, Microtherapeutics was acquired by ev3; Covidien was acquired by Medtronic; Target was acquired by Boston Scientific, which was later acquired by Stryker; and Micrus was acquired by Codman serving as a subsidiary of Johnson and Johnson.

### Practice share of aneurysm treatment

The earliest techniques of endovascular embolization were developed by neurosurgeons and neuroradiologists in the 1960s and 1970s for the treatment of “inoperable” cerebrovascular lesions (7). Since this nascent period of neurointervention, the overwhelming majority of aneurysm embolizations have been performed by interventional neuroradiologists and endovascular neurosurgeons, the former citing their mastery of angiography and image-guided procedural techniques as qualifications, and the latter citing their anatomical expertise and deep understanding of aneurysms (7). For decades, these specialties have worked in tandem to advance the technical feasibility of complex intracranial navigation and aneurysm embolization.

### Early history of neurointervention

Vessel catheterization has become widely implemented for diagnostic and therapeutic strategies in clinical medicine (8, 9). Pioneered by Reverend Stephen Hales in the early 18th century in an equine model, the broad impact of catheterization was recognized when Andre Frederic Cournand, Werner Forssmann, and Dickinson Richards were awarded the Nobel Prize in Physiology or Medicine for their discoveries concerning cardiac catheterization in 1956 (10, 11). Specific to the intracranial circulation, Antonio Caetano de Abreu Freire first described diagnostic cerebral angiography in 1927 for the intended imaging of aberrant vascular patterns surrounding brain neoplasms (this work was published under the *nom de plume* “Egas Moniz,” the name of a 12th century Portuguese nobleman, due to de Abreu Freire's socioeconomic background) (12). He would later go on to win the 1949 Nobel Prize in Physiology or Medicine for his work surrounding frontal leucotomy for psychiatric conditions. Following these landmark advances in vessel catheterization and angiography, Alfred Lussenhop and Alfredo Velasquez reported the first therapeutic catheterization of human cervical vessels in 1964 when they described one case of a ruptured saccular aneurysm of the internal carotid artery (ICA) that underwent technically successful embolization using a 2.5 mm spherical Silastic embolus (13). Despite the eventual poor clinical outcome in the reported patient, this early experience was essential for the development of neurointervention, and was followed by several attempts at improving vessel navigation and minimizing vessel trauma (14). The 1960s also saw the arrival of the first microcatheter, use of magnetic guidance strategies, and a method of aneurysm embolization using detachable magnetic tips with accompanying metal embolic particles (15). In the 1970s, endosaccular balloon occlusion techniques rose to prominence when Serbinenko reported treating over 300 cerebral aneurysms using this technique (16, 17). While several centers and operators advocated for the use of balloon embolization for intracranial aneurysms, this strategy was ultimately deemed unsafe, with high rates of aneurysmal rupture and poor treatment durability (14). It was not until the arrival of coiling that routine endovascular treatment of intracranial aneurysms became a viable technique. Prior to endovascular coiling, endovascular aneurysm treatment primarily involved parent vessel occlusion following balloon test occlusion for aneurysms not amenable to surgical clipping (18, 19).

## Endovascular coiling

The evolution of endovascular devices into those that are available today involved many iterations of treatment strategies with a variety of hypothesized mechanisms of actions for each treatment. The arrival of endovascular coiling marked a major turning point in neurointervention because it achieved durable aneurysm occlusion without overwhelming risk to the patient (14). While coils had already been available for the treatment of various intracranial lesions and parent vessel occlusions, Guglielmi, Vinuela, Sepetka, and Macellari exploited the availability of delivery systems which were smaller than the traditional 5 French and 4 French sizes (including the Tracker microcatheter and Dasher microwire, Target Therapeutics, San Jose, CA, USA) to facilitate intracranial navigation. These navigation tools were paired with soft platinum detachable coils, which evolved into stretch-resistant coils by placement of a suture or wire within the primary coil, to develop endosaccular aneurysm coiling in the early 1990s (20, 21). Their strategy was based on prior microcatheter delivery of coils performed by Sadek Hilal and included positioning the tip of a microcatheter into the neck of a saccular aneurysm and advancement of a platinum coil using a stainless-steel delivery wire (20). This was followed by application of a positive direct electric current to the proximal portion of the delivery wire to initiate electrothrombosis and cause the platinum coil to detach within the aneurysm. The electrothrombosis aspect of their strategy was based on the early work of Sean Mullan at the University of Chicago, who used an open surgical approach to cavernous aneurysms and puncture of the aneurysm with copper wire (22). In their first clinical experience with this strategy, Guglielmi et al. achieved partial or complete aneurysm occlusion in all patients with only one instance of transient neurologic deficit (21). At the time, the prevailing hypothesis was that intra-aneurysmal occlusion was achieved *via* electrothrombosis of negatively charged white blood cells, red blood cells, and blood products with application of a positively charged coil promoting clot formation (22, 23). Later study would confirm that the therapeutic benefit of coiling was achieved due to the space-filling of coils as platinum coils with non-electrolytic detachment had similar rates of efficacy and recurrence (24). Potential mechanisms for preventing aneurysm rupture include slowing flow of blood in and out of the aneurysm to promote clot formation and subsequent intimal growth, as well as other mechanical effects such as flow diversion or biological interaction of the coils with the aneurysm wall.

A major turning point in the use of endovascular coiling was the International Subarachnoid Aneurysm Trial (ISAT) of ruptured intracranial aneurysms published in 2002 that demonstrated improved disability-free survival in aneurysms treated *via* endovascular coiling compared to those treated with surgical clipping (25). This catalyzed a shift away from the “clip-first” approach for the majority of intracranial aneurysms and led to a dramatic increase in the volume of brain aneurysms treated endovascularly. Indeed, from 2004 to 2014 a total of 79,627 intracranial aneurysms were treated *via* endovascular coiling while 42,256 were treated surgically in the United States, a dramatic transition from the distribution of treatment types prior to the publication of ISAT (1).

Following its integration within clinical neurointerventional practice, endovascular coiling was widely adopted. This was accompanied by device developers designing bioactive coils such as the Matrix 1 and Matrix 2 detachable coils (Boston Scientific/Target, Fremont, CA, USA) and the Cerecyte coils (Micrus Endovascular, San Jose, CA, USA). Biologically inert hydrogel-coated coils including the HydroCoil Embolic System (Microvention, Aliso Viejo, CA, USA) were later developed in an attempt to better-occupy the space within the aneurysmal sac (Table 1) (26, 27). Coated and modified coils continue to be of tremendous utility for neurointerventionalists. Though randomized trials comparing recurrence rates of aneurysms treated with hydrogel-coated coils to bare platinum coils initially provided mixed results, more recent level 1 evidence has shown that there may be a benefit to hydrogel coils in ruptured aneurysms compared to bare platinum coils (28–31). Unfortunately, a similar benefit was not observed for bioactive coils. While preclinical data for the Matrix coils, which are coated with a bioabsorbable polyglycolic/polylactic acid polymer designed to facilitate hyperplasia, suggested that there may be therapeutic benefit to these coils, subsequent randomized study and long-term follow-up revealed that there was no clinical benefit to using these coils over bare platinum coils (32–36). Bioactive coils also carry the disadvantages of increased coil stiffness, greater friction between coils, and higher costs than bare platinum coils. Later manufacturers revisited bare platinum coils with different detachment technologies or space filling properties, leading to the arrival of coils including the Axium coils (Medtronic Neurovascular, Irvine, CA, USA), PC400 coils (Penumbra, Alameda, CA, USA), Barricade coils, and Optima coils (Balt, Montmorency, Fr) (Table 1).

Endovascular coiling has several limitations. These include aneurysm recurrence, coil extrusion and migration, limited use for wide-necked saccular aneurysms, challenges associated with aneurysms containing arterial branches, and difficulty with catheter positioning for distal aneurysms (37–40). These limitations would be addressed by subsequent innovations in device and delivery system designs. Despite the limitations of coiling, it continues to be used frequently in acutely ruptured aneurysms, as well as in patients who cannot tolerate antiplatelet therapy.

## Coiling with adjuncts

One major limitation of stand-alone coiling is its inability to achieve a high degree of intra-aneurysmal coil filling in large aneurysms (aneurysms with dome-to-neck ratios > 2.0) (41). The first strategy to address this issue was balloon-assisted coiling, introduced by Moret et al. (42). In this technique, a non**-**inflated balloon catheter is guided across the neck of the aneurysm and inflated during coil deployment to promote adequate coil placement and prevent coil herniation into the parent artery (43, 44). After testing for coil stability with the balloon deflated, the coil is detached, reinflated, and the delivery wire withdrawn. The balloon is withdrawn following deployment of the coil mass. While this technique achieves higher rates of aneurysm occlusion than possible with stand-alone coiling in larger, wide-necked aneurysms, it carries risks including clot formation, vessel rupture, vessel dissection, and intraprocedural aneurysm rupture (41, 42). Balloons had previously been used for temporary occlusions during endovascular coiling, where intermittent coiling could be performed during periods of balloon inflation and transient occlusion. The advent of vessel remodeling with balloons led to the proliferation of both single and double-lumen balloons for endovascular aneurysm treatment (45). Available single-lumen balloons include the HyperGlide and HyperForm balloons (Medtronic Neurovascular, Irvine, CA, USA) and the TransForm balloon (Stryker Neurovascular, Fremont, CA, USA). Double-lumen balloons include the Scepter balloon (Microvention Terumo, Aliso Viejo, CA, USA) and Eclipse balloon (Balt, Montmorency, France). Double-lumen balloons have advantages including increased torquability from the additional lumen that accommodates a second microwire and greater support for navigation through tortuous vessels (46). Balloon-assisted vessel remodeling for coiling continues to be used in both unruptured and ruptured wide necked aneurysms. Intermittent balloon inflation is also performed in the management of intraprocedural ruptures.

Another technique developed to address the technical challenges associated with large, wide-necked aneurysms or aneurysms with characteristics unfavorable for stand-alone coiling is stent-assisted coiling (41). In this strategy, an intravascular stent serves the same role as the balloon employed in balloon-assisted coiling: to ensure proper coil positioning and prevent coil extrusion. Unlike balloon-assisted coiling, the stent is not withdrawn and provides permanent stabilization to the coil mass. In this technique, the coiling microcatheter is placed within the aneurysm either prior to stent deployment or after stent deployment, at which point the coils are placed directly into the aneurysm. The retained stent serves as an additional scaffold for endothelial healing, which can promote repair of the parent vessel as well as exclude the aneurysm, and redirects flow away from the aneurysm and diseased vessel (47). The first case of stent-assisted coiling was described by Higashida et al. (48), and demonstrated a successful treatment of a fusiform basilar aneurysm using an intravascular cardiac stent to act as a scaffold through which coiling could be performed. For the next half-decade, stent-assisted coiling was performed using cardiac stents. Use of cardiac stents in the cerebral circulation was limited by their inflexible design, which precluded safe navigation in more distal cerebral vessels. This changed with the arrival of the Neuroform stent (Boston Scientific/Target, Fremont, CA, USA) in 2002, which was the first intravascular stent designed specifically for cerebral vessels (49). The Neuroform was a self-expanding nitinol stent with an open-cell design with mesh wide-enough to allow coil delivery through its struts (50). While the open-cell design allowed for re-entry and placement of additional coils in the event of aneurysm recurrence, its non-coated nitinol design made it susceptible to galvanic corrosion from a nitinol stent and platinum coils making contact, destabilizing the stent-coil construct, and making stent migration more common in the early Neuroform experience (51–53). The development of the Neuroform stent was followed by the Enterprise stent (Cordis Neurovascular, Miami Lakes, FL, USA) in 2007 (54). Unlike the Neuroform, the Enterprise has a closed cell design that enables partial deployment and recapture and redeployment to achieve satisfactory positioning and coil retention, and is coated by an insulating polymer (Parylene C) that improves the stent's resistance to galvanic corrosion (54). The Enterprise stent was followed by the Low-Profile Visualized Intraluminal Support (LVIS) stent and LVIS Jr. stent (Microvention, Aliso Viejo, CA, USA), which received FDA approval in 2018. The LVIS and LVIS Jr. are both closed cell stents with a braided design, which allows for the use of a “shelf” technique to shape the stent into a configuration that may obviate the need for excessive stent manipulation (55). The braided design also allows for more facile stent conformation to tortuous vessel walls. The newest generation LVIS stent, the LVIS EVO, has enhanced visibility and features smaller cells but has not received approval in the United States. Shortly after approval of the LVIS stents, the Neuroform Atlas (Stryker Neurovascular, Fremont, CA, USA) received approval in 2019 and, like the first generation Neuroform stent, is self-expanding and does not foreshorten, making it easier for operators to gauge the length of the stent (56). Unlike the braided design of other current-generation stents, the Neuroform Atlas is laser-cut and can be delivered *via* a 0.0165-inch microcatheters, facilitating navigation in distal vessels. Despite the success of stent-assisted coiling in treating wide-neck aneurysms and other aneurysms that were not previously amenable to endovascular treatment with stand-alone coiling, technical problems such as stent malpositioning and uncommon dislodgements can occur and have a detrimental effect on treatment efficacy and safety (57). The magnitude of these challenges is amplified in aneurysms with particularly wide necks and those located at bifurcation points where stent deployment is technically difficult. To circumvent these challenges, neurointerventionalists utilized multi-stent configurations. Chow et al. demonstrated the original Y-configuration double stent technique in 2004 using two Neuroform stents in a Y-configuration to treat a basilar tip aneurysm (58). This technique has been adopted by many clinicians, who use various combinations of endovascular stents and positioning strategies to create two-stent construct (59). Other complex and double-stent coiling configurations including X-stenting and T-stenting have been employed to treat bifurcation aneurysms at the basilar tip, internal carotid artery terminus, anterior communicating artery, and middle cerebral artery bifurcation (60–62). T-stenting can also be achieved with a single stent placed horizontally *via* the posterior communicating arteries across the neck of a basilar tip aneurysm (63). While stent-assisted coiling strategies can be used in unruptured and ruptured wide-neck aneurysms, the placement of an intracranial stent necessitates dual antiplatelet therapy, which may worsen bleeding from the vessel or from any subsequent indicated surgical interventions including ventriculostomy, craniotomy, or hemicraniectomy (64).

While stent-assisted coiling using multi-stent configurations addressed many of the challenges associated with wide-neck aneurysms found at bifurcations along the circle of Willis, specialists sought to design more-selective devices for wide-neck bifurcation aneurysms. No device designed specifically to facilitate coiling of wide-neck aneurysms was approved by the FDA until the PulseRider device in 2018 (Pulsar Vascular, San Jose, CA, USA), though devices with similar properties, the pCONUS, pCONUS 2, pCONUS 2-HPC (Phenox, Bochum, Ger), had been available in Europe for several years. The PulseRider device has a distal frame that can be placed across the aneurysm neck, within the aneurysm, or in a combination, which allows for device conformation to vessel walls when deployed and preserves vessel patency without necessitating catheterization of branch vessels (65). The PulseRider device provides stability to the coil mass, promotes hemodynamics favorable for healing of the diseased vessel, and serves as a scaffold for endothelialization (66). Despite these perceived procedural advancements, outcomes when using this device were not necessarily better than those achieved with multi-stent configurations in stent-assisted coiling, leading experienced operators to wonder if these devices became available at an impractical time, as clinicians were skilled using stent-assisted coiling and alternative devices such as the Woven Endobridge (WEB, Microvention-Terumo, Aliso Viejo, CA, USA) were becoming increasingly available (66–68). However, for small aneurysms or aneurysms greater than 12 mm in diameter, PulseRider may be a treatment option with coils.

## Liquid embolics

Liquid embolization is most commonly utilized to treat arteriovenous malformations and fistulae, but has also been employed for intracranial aneurysms (69). Around the time of the first stand-alone coiling procedures, some clinicians began utilizing liquid embolization materials in tandem with other space-occupying embolic materials. The use of this strategy reflected an improved understanding of the mechanism of aneurysm exclusion from the intracranial circulation, as it did not fit the electrothrombosis model that had previously garnered support in the neurointerventional community. In the 1990s, Taki et al. treated 19 giant aneurysms using combinations of detachable balloons, occlusion coils, and ethylene vinyl alcohol copolymer liquid (70). A report published soon after by Kinugasa et al. reported direct infusion of cellulose acetate polymer solution to treat seven aneurysms located in the internal carotid artery (ICA) (71). This solution utilized by Kinugasa et al. had the benefit of hardening completely to achieve aneurysm exclusion. However, there were concerns regarding the generation of emboli during this process, as well as the application of dimethyl sulfoxide directly into the cerebral vasculature, leading to temporary abandonment of these techniques (72). Later study in 2000 showed that intrasaccular liquid embolization could be performed safely with protective devices in tandem with a new generation of liquid embolic materials (73). This new generation of materials included a more viscous Onyx HD-500, which is composed of an ethylene vinyl alcohol copolymer dissolved in a DMSO solvent. In 2004, results from the Cerebral Aneurysm Multicenter European Onyx (CAMEO) trial were published and found that Onyx HD-500 embolization of aneurysms that had failed prior treatment was superior to reported rates of coil occlusion for similar aneurysms (74). Additional study demonstrated the use of protective devices such as stents while performing liquid embolization was warranted to achieve good clinical outcomes (75).

Despite favorable results with this technique when performed by proficient operators, the complexity and tedious nature of the procedure contributed to its abandonment. Repeated balloon inflation and deflations, paired with the need for delicate “seal tests” where contrast is gently injected into the aneurysm, leads to prolonged procedures, particularly in cases where contrast injection disrupts the embolic material (76). Balloon inflation must be limited to minimize the risk of cerebral ischemia in the vessel distal to the balloon, but inadequate inflation also may risk emboli and improper precipitation of the liquid embolic material. Migration of the balloon in serial deflation/reinflation cycles may require retrieval and redeployment, further increasing the duration of the procedure. It has been suggested that in the modern era of flow diversion and other treatments, liquid embolization as a viable treatment strategy for patients with incomplete aneurysm occlusion and persistent high-risk features, as well as patients with nickel allergies, though the veracity of nickel allergies leading to relevant complications has been recently called into question (77).

Unlike the clear shift from stand-alone coiling to stent-assisted coiling for many aneurysms, no study initially demonstrated inferiority of intrasaccular liquid embolization compared to newer strategies. Long-term follow-up did ultimately demonstrate suboptimal durability of this treatment strategy (75). Intrasaccular liquid embolization was adopted at a higher rate at our center compared to other centers in the United States. In our institutional experience, intrasaccular liquid embolization was commonly selected prior to the flow diversion era and provided favorable safety and efficacy in large and giant aneurysms with unfavorable anatomical configurations. In the current age of flow diversion and intrasaccular flow disruption, intrasaccular liquid embolization has few indications and is no longer commercially available despite its ability to theoretically fill 100% of an aneurysm, provide a surface for endothelialization, and its use to occlude giant aneurysms (78).

## Flow diversion

Intracranial stents utilized for stent-assisted coiling had some limited flow-diverting properties, with some operators occasionally using these stents as stand-alone treatments or exploiting these properties to improve treatment efficacy (79, 80). Preclinical studies established that stents with a higher degree of metal coverage and lower porosity could increase the degree of flow directed away from the aneurysm without measurably increasing the risk of side branch occlusion or thrombosis, which was a major concern in the early era of flow diversion (81, 82). While several neurovascular innovations had generated intrigue within the neurointerventional community, there was a particularly high amount of excitement surrounding flow diversion technologies in light of promising results from international experiences with the Pipeline Embolization Device (PED) (Medtronic Neurovascular, Irvine, CA, USA) (83–85). This early enthusiasm was appropriately dampened as skilled operators reported concerns regarding hemorrhagic events and in-stent thrombosis, the latter highlighting the need for compliance to antiplatelet therapy (86, 87). However, large multi-center studies would go on to show very favorable safety and efficacy for the PED (85, 88).

As operators became more proficient with flow diverting devices, indications for the PED expanded to include segments of the ICA distal to the terminus, and off-label use of the PED has been seen in virtually every cerebrovascular territory (3, 89). The original PED is a self-expanding cylinder composed of 36 cobalt-chromium strands and 12 platinum-tungsten strands woven in a 3:1 ratio (90). The next generation PED was the Pipeline Flex, and had an improved delivery microcatheter that enabled recapture and resheathing of the PED, facilitated redeployment, and replaced the capture coil with double Teflon enveloping sleeves to eliminate challenges with expansion of the device (91). The Pipeline Flex received FDA approval in 2015. Most recently, Pipeline Flex-Shield received FDA approval in 2021 and has a surface treatment of phosphorylcholine to reduce in-stent thrombus formation and stenosis (92). The Pipeline Vantage device, which includes modifications such as a thinner frame, higher porosity, and a novel microwire design, has not received FDA approval but is expected to follow the Pipeline Flex-Shield in the United States. Besides the Pipeline flow diverters, several other flow diverters are available, though these are less frequently used. The Surpass Streamline and Surpass Evolve stents (Stryker Neurovascular, Fremont, CA, USA) received approval in 2018 and 2020, respectively. The Surpass Streamline stent is composed of either 72 or 96 cobalt-chromium wires depending on stent size, while the Surpass Evolve is composed of 64 cobalt-chromium wires, giving both models higher mesh coverage than other flow diverters, which may theoretically accelerate vessel healing and aneurysm occlusion (93). The Surpass Evolve has a higher braid angle to improve wall apposition, a problem that hindered the first generation Surpass stents, and is also deliverable with smaller microcatheters (0.027-inch) than the first-generation models (47). Another flow diverter, the Flow Re-Direction Endoluminal Device (FRED) (Microvention, Aliso Viejo, CA, USA), is composed of a self-expanding braided nitinol mesh with an inner wire flow diverter for a total of 66 wires, and received FDA approval in 2019 (94). The FRED Jr followed soon after, and is dedicated to treating aneurysms in small vessels given its ability to be delivered with a 0.021-inch microcatheter compared to the 0.027-inch microcatheters used for PEDs (95). Though not approved by the United States FDA, recent flow diverters approved by European regulatory agencies provide a comparative advantage in their ability to be delivered with even smaller microcatheters. The Silk flow diverter and Silk Vista Baby (Balt Extrusion, Montmorency, Fr) are deliverable *via* 0.021- and 0.017-inch microcatheters and provide easier access to distal aneurysms than those delivered by larger-diameter microcatheters (96). The FRED X was recently released and also is optimized for smaller, more distal vessels and features a protective hydration layer to minimize thrombogenicity.

Technically, deployment of a flow diverter occurs in a distal to proximal fashion with the delivery catheter in the vessel distal to the aneurysm (97). The device is then unsheathed across the aneurysmal neck. Flow diverters are currently re-sheathable to allow retrieval and redeployment into the optimal position. To provide the appropriate coaxial support necessary for controlled deployment of flow diverters, a new type of delivery catheter was developed. In previous coiling-based procedures, a rigid guide catheter was typically positioned in the cervical ICA, from which a microcatheter was navigated distally. As the microcatheter moved more distally, operator control gradually decreased due to the lack of support. A new generation of catheters (intermediate catheters) was smaller than the sheath but larger than the microcatheter. These could be navigated from the cervical ICA up to and into the circle of Willis, and provide more rigid proximal support for microcatheter navigation and device deployment (98, 99). Intermediate catheters include the Navien intracranial catheter (Medtronic Neurovascular, Irvine, CA, USA), the Sofia catheter (Microvention-Terumo, Aliso Viejo, CA, USA), and the Outreach distal access catheter (Stryker Neurovascular, Fremont, CA, USA) (100).

Flow diverters, which are high pore devices that cover 30–40% of the vessel wall with metal, depending on the size and number of wires and the angles of the braid, achieve aneurysm occlusion in three phases (47). First, there are acute hemodynamic changes as blood is diverted away from the aneurysm (47). This is followed by stable thrombus formation, which occurs due to platelet aggregation within the aneurysm, producing an effect similar to the initial electrothrombosis hypotheses that surrounded coiling in the 1990s. Finally, endothelialization occurs, with the flow diverter serving as a scaffold for this process and facilitating the final exclusion of the aneurysm (47). Success is achieved by placing enough flow diverters across the neck of the aneurysm to restrict flow in and out. At times, this may require placement of two or three additional devices through the first to achieve adequate flow diversion and scaffolding for the endothelium. As a consequence of this process, recurrence after angiographic cure of a flow diverted aneurysm is exceedingly rare (101). More broadly, the arrival of flow diversion removed the need to enter the aneurysm itself during treatment, obviating the inherent risks of these maneuvers. By 2016, over 70% of procedures at our center involved placement of either a flow diverter or stent, demonstrating the shift toward utilizing scaffolds to facilitate aneurysm exclusion. A drawback to flow diversion and stenting is the requirement for dual antiplatelet therapy (DAPT) to avoid thromboembolic complications. Many operators elect to carefully titrate and measure antiplatelet activity with assays including the VerifyNow (Werfen, Bedford, MA, USA) assay, which may help mitigate the risk of such complications (102).

## Intrasaccular flow disruption

Flow diverting stents proved to be versatile devices and are capable of reaching durable aneurysm cure by promoting healing of the diseased parent vessel (101, 103). Drawbacks to flow diversion including the need for DAPT to minimize thromboembolic complications, as well as relatively poor efficacy when employed to treat wide-neck bifurcation aneurysms compared to side-wall aneurysms (104). These difficulties, paired with technical difficulties associated with complex stent configurations, led to the development of a new class of endovascular aneurysm treatment. Intrasaccular flow disrupting devices were first utilized in 2010, but only recent became available in the United States (2018) following the Woven Endobridge-Intrasaccular Therapy (WEB-IT) prospective study (66, 105). The early international adoption of this device allowed for substantial detail to be shared with operators seeking to use the WEB in the United States (106). For example, the WorldWideWEB consortium has a decade of data corresponding to patients treated using the WEB. This data has recorded trends toward more frequent use of the WEB in off-label applications and improved immediate and long-term angiographic cure, with no variation in complication rate or mortality (106). Study has also shown that the WEB device achieves similar rates of angiographic occlusion at 1 year follow-up in ruptured aneurysms as is achievable in unruptured aneurysms, and similar safety and efficacy to other devices for ruptured aneurysms (107). This was an important improvement compared to its intrasaccular predecessor, the Medina embolization device (Covidien, Irvine, CA), which had poor rates of long-term efficacy and was highly-dependent on precise device sizing for a given aneurysm and adequate accompanying coiling (108).

Not unlike the first phase of aneurysm healing following flow diversion, intrasaccular flow disruption involves immediate alteration of the hemodynamics within the aneurysm (109). These devices are placed entirely within the aneurysm, have no extension into the parent vessel, and cover the aneurysm wall and neck with a high-density metallic mesh composed of nitinol wires at the base with a platinum core in an ellipsoid shape (109, 110). The first generation of WEB devices were composed of a double mesh layer at the base, but have since been modified to a single layer of mesh. Because of the lack of intrusion into the parent vessel, no antiplatelet therapy is required for patients who undergo WEB treatment, though some operators elect to prescribe a brief regimen of Aspirin or DAPT prior to WEB embolization in the event a stent needs to be placed to preserve patency of the parent vessel (111). If a stent is not required, the P2Y12-inhibiting medication is discontinued. Another advantage of the WEB is its application for bifurcation aneurysms that contain eloquent perforating arteries without compromising supply through these crucial vessels. It is believed that obliteration following disruption of flow within the aneurysm occurs because of surface tension causing rapid stasis of blood, leading to thrombosis and occlusion of the aneurysm (109). Currently, the WEB is approved for use at bifurcation points of anterior communicating, middle cerebral, internal carotid, and basilar arteries (111). The WEB has been widely adopted by many centers and has replaced several previous devices (such as Onyx HD-500, PulseRider, stent-assisted coiling) utilized in the treatment of wide neck bifurcation aneurysms. Despite the promise of WEB devices, there are some challenges in deployment within irregular and multilobular aneurysms, and the current strategy for these aneurysms involves targeting the primary lobe to treat all associated lobes (111). This may be technically challenging, and the next generation of aneurysm treatment devices would do well to address this area of aneurysm treatments. Like coiling, WEB placement also requires entry into the aneurysm and therefore carries a small risk of intraprocedural aneurysm rupture prior to partial opening of the device, which expands and softens upon partial opening. Finally, the WEB relies on lateral expansion in its deployment, and is not suited for small (<3 mm) or large (>10 mm) aneurysms.

Following the WEB, the Contour neurovascular system (Cerus, Concord, CA, USA) was developed recently with the aim of reducing challenges associated with prior generation endosaccular devices. Specifically, it expands to fill the proximal half of the targeted aneurysm in a cup-like shape (112). This allows for sizing to be performed specifically based on the aneurysm diameter and width. Early clinical experience with this device suggest favorable rates of safety and efficacy, though its recent introduction precludes the availability of robust long-term follow-up data (113).

## Antiplatelet therapy

The majority of aneurysm treatments performed at our center and others involves the placement of either a flow diverter or stent. However, treating aneurysms with these endothelialization scaffolds requires peri-procedural DAPT to avoid thromboembolic complications. While DAPT regimens most commonly comprise Aspirin and Clopidogrel, some operators prefer to combine Prasugrel, Ticagrelor, or Cangrelor with Aspirin (114). Some report monotherapy with Aspirin or P2Y12 inhibitors, though this is a far less common approach (114). The use of antiplatelet therapy in acutely ruptured aneurysms continues to be an area of contention in neurointervention, requiring operators to balance the risk of procedure-related thromboembolism in these situations with risk for more severe bleeding (115–117).

## Conclusion

As described in this review, the evolution of endovascular aneurysm treatment has been an iterative process, with new waves of devices addressing technical challenges of the prior generation. These device-level innovations have been accompanied by other advances that have improved the safety and efficacy of endovascular brain aneurysm treatment. Alternate access to the intracranial circulation using transradial approaches have become favored by many operators compared to conventional transfemoral approaches (118). Novel microcatheters and microwires have enabled operators to catheterize distal vascular territories that were previously inaccessible (119). A growing understanding of appropriate uses of antiplatelet therapies have assisted in mitigating both hemorrhagic and ischemic complications after certain embolization procedures (120–122). Finally, optimization of follow-up protocols after aneurysm treatment have eliminated low-value follow-ups, minimized patient risks associated with cerebral angiography, and mitigated the perception of unnecessary barriers to appropriate care (123, 124). Future advances in endovascular brain aneurysm treatment devices may exploit our understanding of the biology of aneurysm healing to apply active protein coatings on devices and promote intra-aneurysmal healing (125, 126).

## Author contributions

Study conception and manuscript writing: DL and CM. Critical revision and final approval: DL, AH, AS, SC, JO, AC, AV, AK, CD, DC, and CM. All authors have read and approved the submitted manuscript.
